# ^18^F-FDG PET/CT as a potential valuable adjunct to MRI in characterising the Brodie’s abscess

**DOI:** 10.2349/biij.6.3.e26

**Published:** 2010-07-01

**Authors:** F Fathinul, AJ Nordin

**Affiliations:** 1 Damai Service Hospital, Jalan Ipoh, Kuala Lumpur, Malaysia; 2 Faculty of Medicine and Health Sciences, Universiti Putra Malaysia, Selangor, Malaysia

**Keywords:** ^18^F-FDG PET/CT, Brodie’s abscess, penumbra

## Abstract

Chronic osteomyelitis (Brodie’s abscess) is essentially a problem of diagnosis, and there may be considerable difficulty in distinguishing it from other benign and malignant bone lesions. Early diagnosis of Brodie’s abscess is deemed important as the disease has a good curative potential following an appropriate antibiotic treatment. Of late, PET/CT using ^18^F-FDG is taking a centre stage in the imaging of bone infection though documentation on its role in characterising the feature of Brodie’s abscess is exceedingly scarce. On the other hand, it is well known that MRI imaging plays a very important role in distinguishing abscess loculation from malignancy. The authors present the case of a 13-year-old boy with pain in the right heel for few months. Radiograph of the right foot revealed a lucent focus with sclerotic margin in the right calcaneum. MRI T1-weighted images were inconclusive of penumbra sign to characterise abscess cavity due to the small volume lesion. Whole-body ^18^F-FDG PET/CT scan showed multiple small avid lesions at the margin of the sclerotic rim in the right calcaneum. Final diagnosis of Brodie’s abscess with Klebsiella culture was confirmed via bone debridement.

## INTRODUCTION

Brodie’s abscess is a rare lesion manifesting as a chronic form of intraosseous osteomyelitis surrounded by dense fibrous tissue and sclerotic bone [1]. It is predominantly reported in young male with unfused epiphyseal plate typically presenting during the second decade of life. The pathogenesis is due to an insidious bacteremia with septic emboli to normal or minimally traumatised bone as a result of an inadequately treated osteomyelitis. Radiography of Brodie’s abscess often defines a localised area of radiolucency with surrounding sclerosis but most of the lesions are imperceptible in the early stage. Pathologically, the wall of the abscess contains large amounts of granulation tissue, accounting for pronounced rim enhancement on contrast-enhanced T1-weighted MRI image with outer layer of hypointensity of which configurations are largely referred as penumbra sign.

The central portions are mainly constituted of necrotic fluid and pathologic organisms [1]. Grey et al. stated that penumbra sign has an accuracy of 99% in diagnosing chronic osteomyelitis and differentiating it from malignancy [1]. The advancement in PET/CT technology using ^18^F-FDG tracer as glucose analog provide a considerably good complementary functional biomarker in defining the chronic osteomyelitis and is a highly sensitive and specific method for the evaluation of chronic infection in the axial and appendicular skeleton in patients with trauma [2]. Furthermore, in certain circumstances, PET/CT is exceptionally effective in delineating inflammatory process in the bone where MRI image-characterisation is degraded by bone implants or due to small volume lesion [2]. The authors highlight the importance of ^18^F-FDG PET/CT utility as a potential adjunct to MRI in characterising Brodie’s abscess in a 13-year-old boy in whom diagnosis was proven by isolation of Klebsiella organism culture via bone debridement.

## CASE REPORT

**Figure 1:**
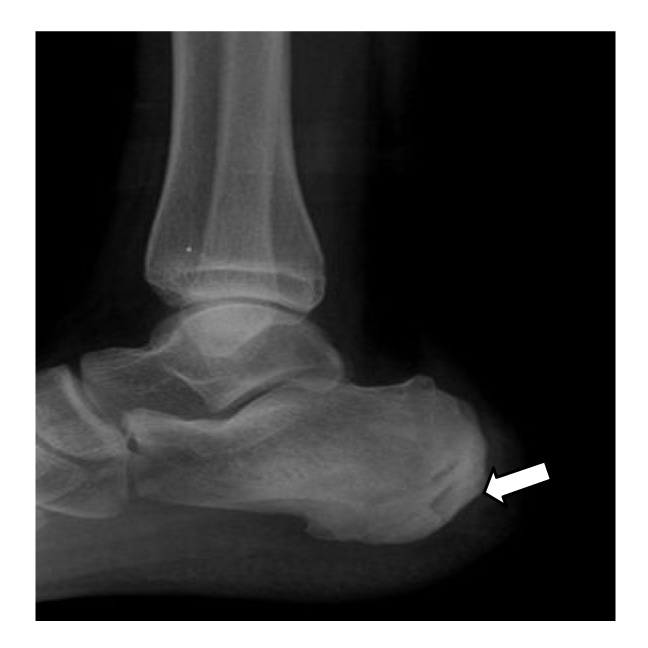
Lateral view of the right ankle shows a small lucent lesion within the calcaneum (arrow). [Image courtesy of Prince Court Medical Centre, Kuala Lumpur, Malaysia ].

**Figure 2:**
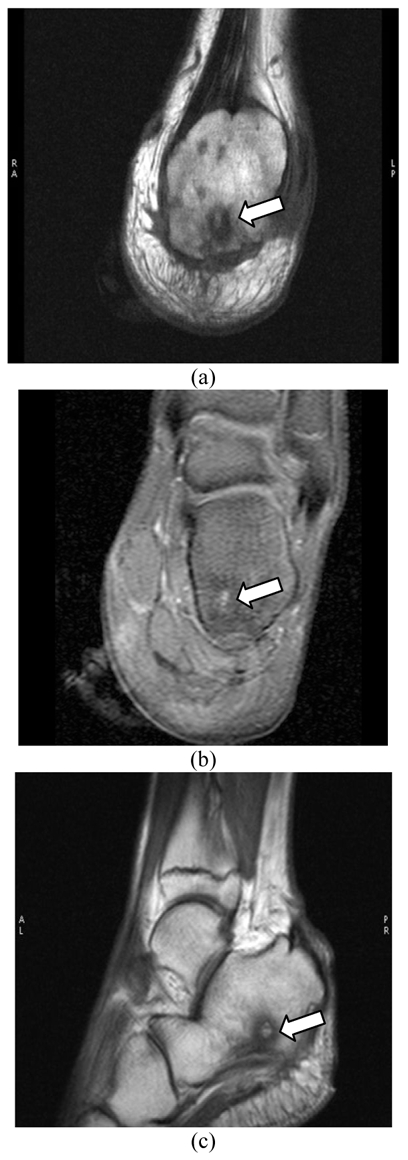
(a) T1SE weighted coronal image of the right ankle displays focus of intermediate intensity intraosseous lesion surrounded by rim of low intensity (arrow); (b) T1SE Fat SAT shows high-intensity focus with the outer layer of low attenuation signal (arrow); (c) T1SE Fat SAT post Gadolinium sagittal image shows no significant rim of enhancement (arrow). Image courtesy of Prince Court Medical Centre, Kuala Lumpur, Malaysia.

**Figure 3:**
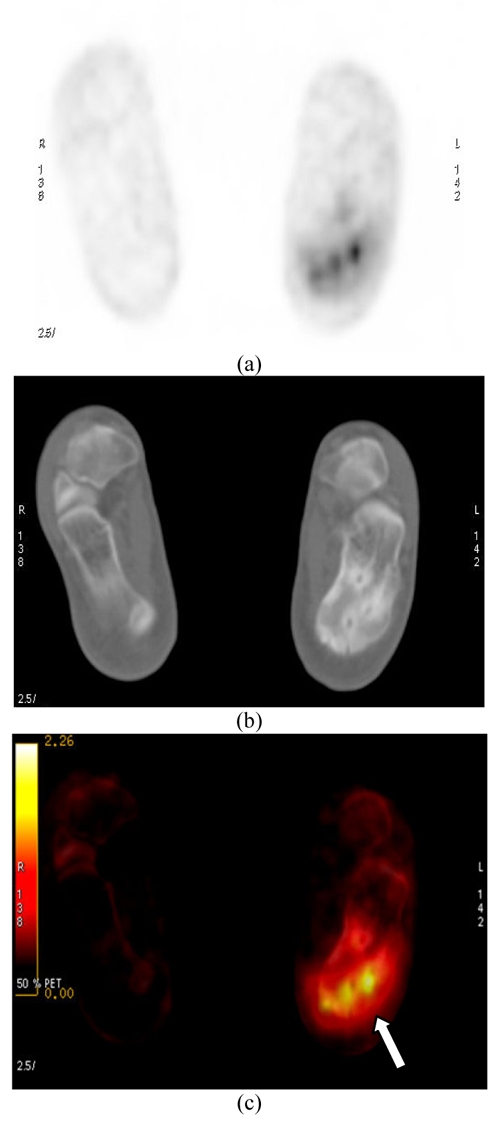
(a) Coronal PET image showing foci of avid ^18^F-FDG intensity signal lesions within the right calcaneum; (b) Coronal low-dose CT image showing foci of low attenuation lesions with rim of sclerosis; (c) A combined PET/CT image displays foci of avid ^18^F-FDG uptake at the sclerotic rims of the right calcaneum extending to the adjacent inferior cortex (arrow).

A 13-year-old boy presented with a history of right heel pain for a few months. His right foot was swollen and tender. He recalled an accidental step on a rusty nail two years back following which he had pain and swelling. The symptoms subsided following courses of antibiotic treatment. The recent radiography of the right ankle revealed a focus of intraosseous lucency within the right calcaneum (Figure 1). MRI study was inconclusive of the penumbra sign as seen on T1-weighted sequences to characterise abscess cavity given the small volume of the lesions (Figure: 2a-c). Co-registered PET/CT images of the right calcaneum displayed multiple foci of ^18^F-FDG avidity at the margin of the sclerotic margin consistence with granulation tissue formation (Figure: 3a-c). Bone debridement yielded the culture of Klebsiella organism and the final diagnosis of Brodie’s abscess was made.

**Figure 4:**
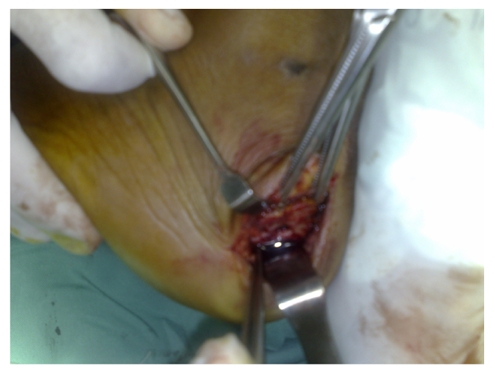
An intraoperative image revealed the site of the abscess loculation within the calcaneum (arrow). Image courtesy of Prince Court Medical Centre, Kuala Lumpur, Malaysia and Salam Specialist Medical Centre, Shah Alam, Malaysia.

## DISCUSSION

This young boy presented with a history of right heel pain for a few months without preceding inciting trauma or symptoms. The past history of trauma to the right heel raises the index of suspicion of an inadequately treated infective bone inoculation which manifested at the present time. Initial radiography of the right ankle was indeed nonspecific as a lucent focus in the right calcaneum may resemble other pathological entity i.e. eosinophilic granuloma or metastatic deposit but these alternate diagnoses can be excluded by the extensive soft tissue inflammation clinically [1]. MRI of the right calcaneum was inconclusive in defining the characteristic penumbra sign of abscess cavity formation on T1-weighted sequences. On ^18^F-FDG PET/CT, foci of tiny rim of avid metabolic activity at the margin of the sclerotic rims points to the formation of granulation tissue [3]. The sclerotic margin invariably represents the sclerosis or involucrum. In this particular case, PET/CT image has successfully defined the disease extent as evident by involvement of the calcaneal cortex. Stumpe et al. concluded that PET using ^18^F-FDG appears to be a highly sensitive method in detecting infective foci in the bone [4]. Histologically, the ^18^F-FDG avidity defines the area of fibroblast proliferation and neovascularisation with mononuclear cell infiltration at the granulation tissue formation whereby these cells utilise most of their energy from the trapped ^18^F-FDG for cells metabolism [1,3]. The reliability of ^18^F-FDG as a biomarker in chronic osteomyelitis is further substantiated by a revelation of an experiment of which findings stated that the maximum uptake of ^18^F-FDG was observed in chronic inflammation far more pronounced than in acute phase [5]. In this regard, it is noteworthy to document that the hybrid PET/CT using ^18^F-FDG is exceedingly a sensitive tool in the imaging of chronic infection given its ability in allowing precise anatomical localisation and optimal characterisation of the abscess formation and its extent by its biological ^18^F-FDG affinity to the chronic inflammatory tissue.

## CONCLUSION

The ^18^F-FDG PET/CT as a form of molecular imaging procedure should be viewed as an important adjunct to an already powerful armamentarium of clinical imaging tools, specifically the MRI imaging. This report underlies the promising method of ^18^F-FDG PET/CT and its further roles as a potential primary workhorse in chronic osteomyelitis in instances where MRI fails to characterise the differentiating feature of abscess formation from malignancy.
